# Substance-Induced Psychosis and First-Episode Psychotic Disorders: Premorbid Risks, Labour Market Outcomes, and Antipsychotic Use

**DOI:** 10.1192/j.eurpsy.2026.10496

**Published:** 2026-07-31

**Authors:** J. Jeyapalan, S. Niemela, H. Taipale

**Affiliations:** 1 Department of Psychiatry, University of Turku, Turku, Finland; 2 Department of Clinical Neuroscience, Karolinska Institutet, Stockholm, Sweden

## Abstract

**Introduction:**

Substance-induced psychosis (SIP) is often considered a transient condition, yet growing evidence suggests its burden may resemble that of first-episode psychotic disorders (FEPD). Comparative data on premorbid risks, labour market outcomes, and pharmacological treatment remain scarce.

**Objectives:**

1. To compare sociodemographic, psychiatric, and work-related characteristics between SIP and FEPD.
2. To evaluate prevalence and patterns of antipsychotic use before and after the first psychotic episode.
3. To assess subtype-specific treatment trends and medications prescribed among SIP patients.

**Methods:**

Nationwide Swedish health registers (2006–2016) identified 7,320 SIP cases (ICD-10 F1X.5, excluding tobacco) and matched them 1:1 with FEPD (F20–F29) by age, sex, and calendar year. Premorbid psychiatric morbidity, self-harm, employment, sickness absence, and disability pension were examined. Antipsychotic use was analysed from three years before until three years after diagnosis, stratified by SIP subtype (alcohol, cannabis, amphetamine, multi-use). Prescriptions were classified by active substance using the ATC system.

**Results:**

SIP patients showed higher rates of ADHD (14.4% vs 8.9%), substance use disorder (68.3% vs 22.3%), and self-harm (22.9% vs 11.3%) compared with FEPD, but lower rates of depression, anxiety, autism, and intellectual disability. They were more often unemployed (24.6% vs 18.6%) and less frequently on sickness absence (8.6% vs 9.9%) or disability pension (18.6% vs 26.3%).

Antipsychotic use peaked 6 months post-diagnosis (23% SIP vs 54% FEPD) and stabilised at 3 years (20% vs 50%). First-year cumulative prevalence: 43% SIP vs 73% FEPD. Cannabis-SIP had the highest prevalence (64.6%), followed by multi-use (50.1%), amphetamine (38.4%), and alcohol (25.2%).

Medication patterns revealed olanzapine as the most prescribed (35% overall; 44% in cannabis-SIP), quetiapine more common in amphetamine-SIP (10%), and risperidone more frequent in FEPD (10% vs 6% in SIP). Use of multiple antipsychotics was higher in FEPD (41.8%) compared to SIP (34.9%).

**Image 1:**
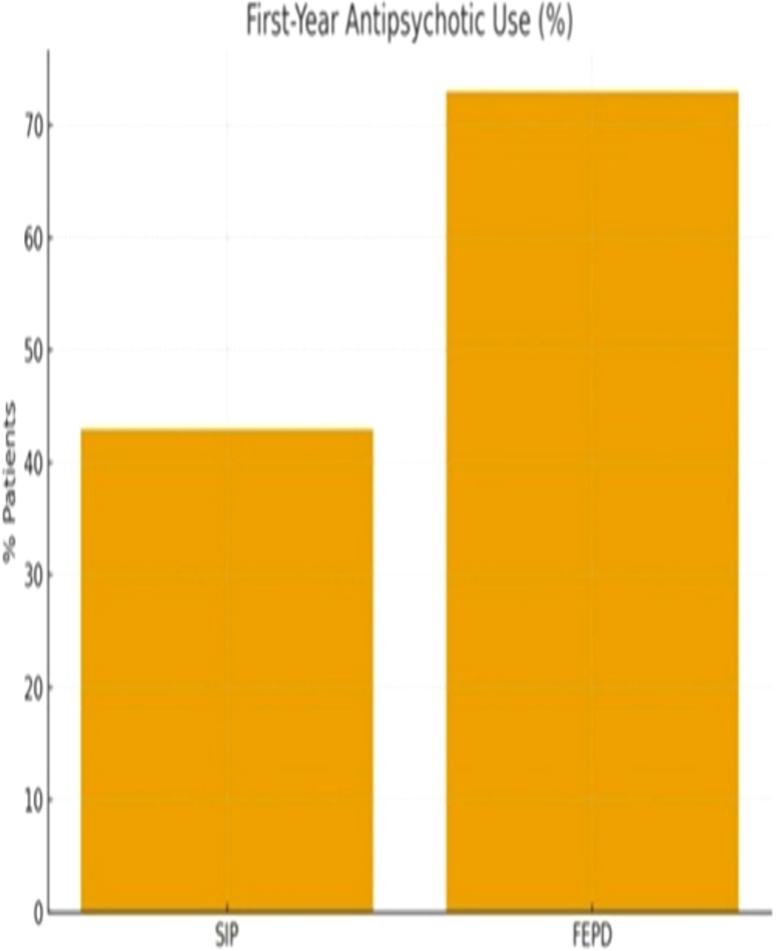

**Image 2:**
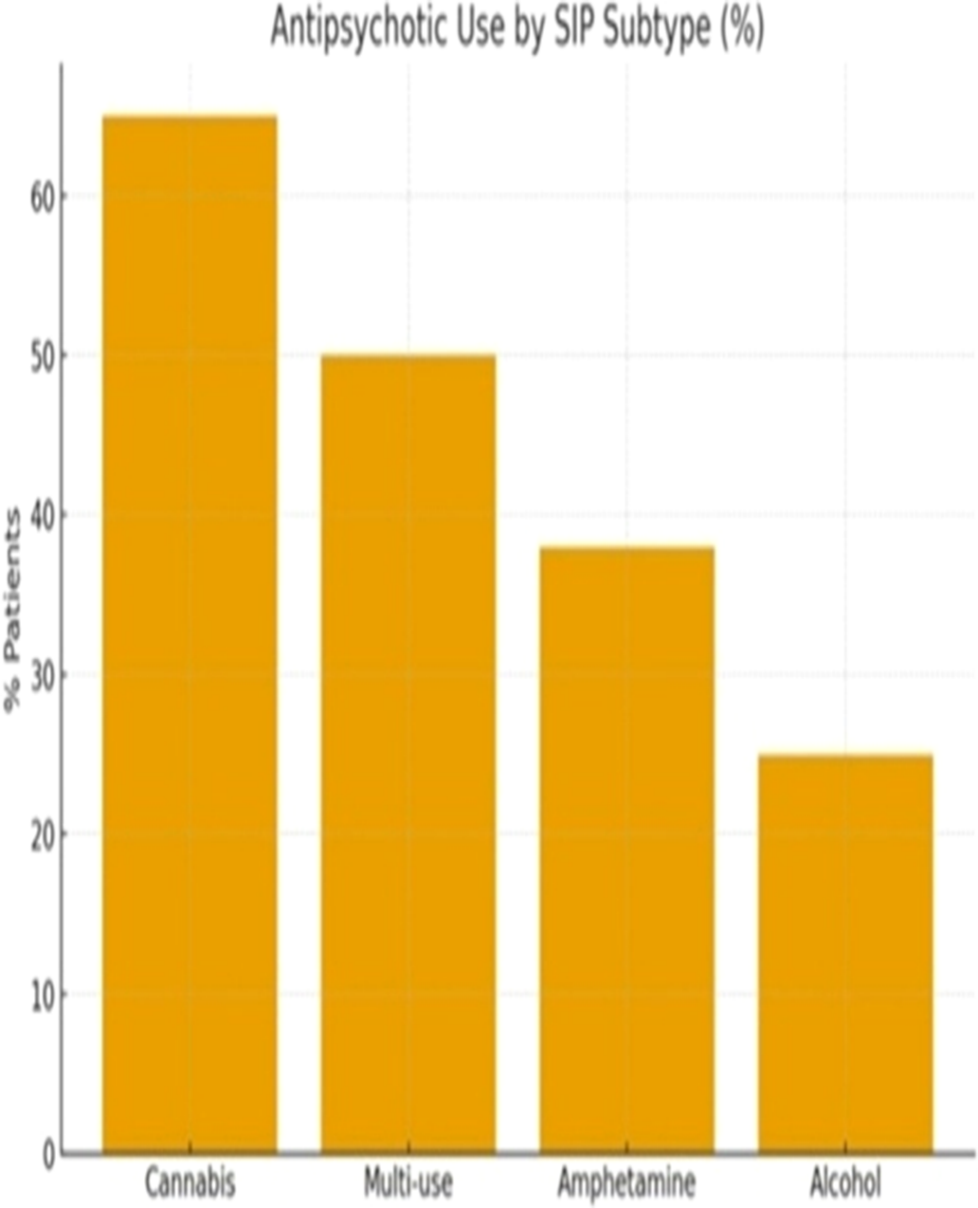

**Image 3:**
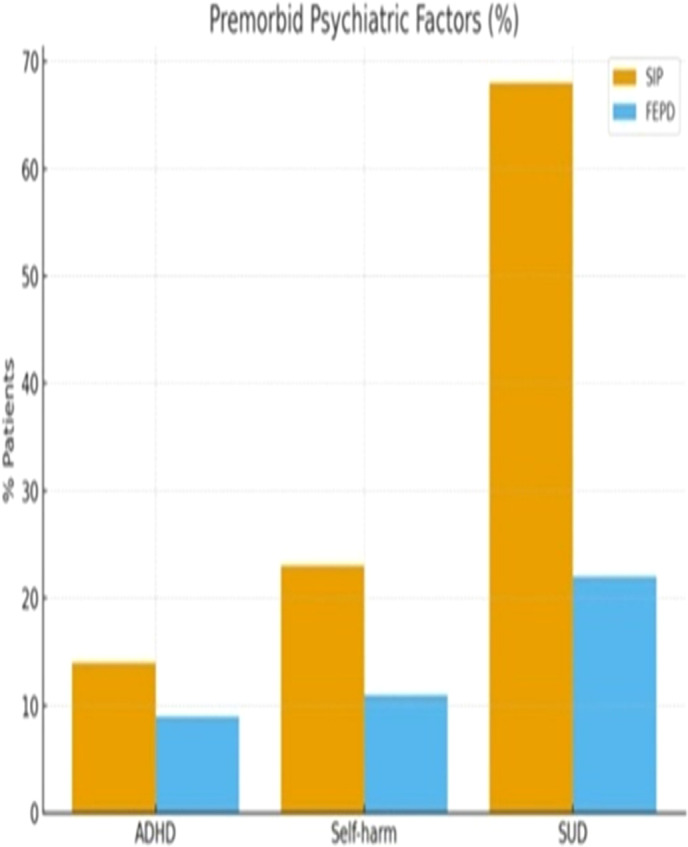

**Conclusions:**

Persons with SIP present distinct premorbid risks, including ADHD, substance use, and self-harm, coupled with labour market marginalisation. Despite assumptions of transience, sustained antipsychotic use was observed, particularly olanzapine in cannabis-SIP and quetiapine in amphetamine-SIP. These findings challenge current diagnostic notions and highlight the need for subtype-specific guidelines to improve clinical management.

**Disclosure of Interest:**

None Declared

